# Low-dose aspirin for the prevention of preterm birth: More questions than answers

**DOI:** 10.1371/journal.pmed.1003908

**Published:** 2022-02-01

**Authors:** Victoria Hodgetts Morton, Sarah J. Stock

**Affiliations:** 1 Institute of Applied Health Research, University of Birmingham, Birmingham, United Kingdom; 2 Usher Institute, University of Edinburgh, Edinburgh, United Kingdom

Preterm birth (birth before 37 weeks gestation) is the leading cause of neonatal mortality, is associated with long-term disability in survivors, and carries a substantial economic burden to healthcare and social services [1]. There is increasing interest in the use of aspirin as a preventative treatment for preterm birth. Low-dose aspirin prophylaxis is well established in women who are at high risk of hypertensive disorders in pregnancy. Meta-analysis of trial data shows that low-dose aspirin taken from early pregnancy is beneficial for reducing the incidence of preeclampsia and its associated complications, including preterm birth [2]. The majority of preterm births associated with preeclampsia are provider initiated, resulting from preterm cesarean section or induction of labour indicated by worsening maternal or fetal condition. Nevertheless, reanalyses of data from trials of aspirin to prevent preeclampsia have also shown small but statistically significant reductions in spontaneous preterm birth (preterm birth preceded by the spontaneous onset of contractions or preterm prelabour rupture of membranes) [3,4]. As spontaneous preterm births are the biggest contributor to preterm birth overall, the question of whether aspirin can be used to prevent spontaneous preterm births has arisen.

There has been little data from primary trials to guide practice in this area. In an accompanying research study in *PLOS Medicine*, Landman and colleagues report on a randomised controlled trial designed to assess the effectiveness of low-dose aspirin in the prevention of preterm birth in women at high risk of preterm birth [5]. Women with a previous spontaneous preterm birth between 22 and 36 weeks gestation (a recognised risk factor for recurrent preterm birth) were eligible to participate in the APRIL (aspirin for the prevention of recurrent spontaneous preterm labour) trial. Participants were randomised to daily aspirin 80 mg or placebo, initiated between 8 and 16 weeks gestation, and continued until 36 weeks gestation. The primary outcome was any preterm birth before 37 weeks gestation (i.e., included both spontaneous and provider-initiated preterm births). Although a small reduction in recurrent preterm birth was observed in women taking low-dose aspirin, this was not statistically significant (21% preterm birth rate in women randomised to aspirin compared to 25% preterm birth in those randomised to placebo). Unfortunately, with 406 participants, the APRIL trial was underpowered to provide a definitive answer for the primary outcome of preterm birth.

The sample size calculation for the APRIL trial was based on a potential 35% relative reduction in the rate of preterm birth (which the authors state was based on the average risk reduction in preterm birth seen in secondary analyses of other trials of aspirin), from a background rate of 36%. This background rate was derived from a trial of progesterone to prevent preterm birth which recruited participants from the United States in the late 1990s, but is higher than that cited in more recent literature, especially if other preterm birth treatments are administered [6,7]. In the APRIL trial, nearly two-thirds of participants were prescribed progesterone, and around 1 in 10 underwent cervical cerclage, which may have contributed to the lower than anticipated recurrent preterm birth rate observed in both arms. At around 17%, the nonsignificant effect size seen in the APRIL trial was smaller than forecast. Taken together, these findings suggest that a trial around 10 times larger than APRIL is required to determine if aspirin can prevent recurrent preterm birth.

The feasibility of carrying out a very large trial in the population of women at risk of preterm birth in the future is uncertain, given that there is already high use of aspirin prophylaxis for preeclampsia in many settings. Although an indication for aspirin was an exclusion criterion for the APRIL trial, the baseline demographics suggest that women were included who would have had aspirin prescribed according to current clinical guidelines in many countries. For example, women with hypertension, kidney disease, systemic lupus erythematosus, or diabetes would be recommended aspirin under the US [2], the United Kingdom [8], Canada [9], Australia and New Zealand [10], and WHO [11] guidance. It seems likely that many more participants had 2 or more “moderate” risk factors for preeclampsia that would also indicate aspirin (e.g., nulliparity, raised BMI, and age >35 years) [2,8–11], especially given the recognised interrelationship between risk factors for preeclampsia and preterm birth.

One approach for future research would be to identify groups of women at risk of specific phenotypes of preterm birth in whom aspirin is most effective. Prespecified subgroup analysis in the APRIL trial [5] hinted that larger effects may be seen with aspirin use in those at highest risk of early spontaneous preterm birth due to previous spontaneous preterm birth at less than 30 weeks gestation. However, without a clear understanding of the mechanism of action of aspirin, and good biomarkers to differentiate different phenotypes of preterm birth, such stratified approaches are likely to be challenging. An alternative approach would be to consider unselected use of aspirin. In a recent large randomised controlled trial in 6 low- and middle-income countries, low-dose aspirin was shown to reduce the risk of overall preterm birth in nulliparous women with singleton pregnancies [12]. The generalisability to high-income settings with lower preterm birth rates and existing clinical guidance for aspirin prophylaxis for preeclampsia remains unknown, but is worthy of future investigation.

A key issue to address in future research is the dose of aspirin that may be most effective. Even for preeclampsia prophylaxis, the optimal dose is uncertain, with ranges from 75 mg to 160 mg daily used [8]. Although safety data regarding the use of aspirin have been generally reassuring, extra surveillance is also required as aspirin use in pregnancy has been associated with increased postpartum bleeding and potentially linked to neonatal intracranial hemorrhage [13]. Indeed, in the APRIL trial, higher mortality was seen in the aspirin group with 6 fetal or neonatal deaths, compared to 2 deaths in the placebo arm [5]. However, any potential increased mortality seems unlikely to be attributable to aspirin per se and most likely reflects the differing background risks of complications between the groups. Women randomised to aspirin had a higher risk of preterm birth than those randomised to placebo due to chance imbalances in the proportion of women with recognised risk factors for spontaneous preterm birth, including a history of midtrimester loss.

The APRIL trial illustrates the need for better understanding of the mechanisms underlying preterm birth to enable development of appropriate and targeted treatments [5]. Until then, more universal and pragmatic approaches may be the best way forward, but these require large-scale evaluation in trials. Unless there are also recognised risk factors for preeclampsia, the answer to the question of whether aspirin can be used to prevent spontaneous preterm births remains uncertain.
